# Antimicrobial Sub-MIC induces *Staphylococcus aureus* biofilm formation without affecting the bacterial count

**DOI:** 10.1186/s12879-024-09790-3

**Published:** 2024-09-28

**Authors:** Raghda Elawady, Aliaa G. Aboulela, Ahmed Gaballah, Abeer A. Ghazal, Ahmed N. Amer

**Affiliations:** 1 Department of Microbiology, Medical Research Institute, Alexandria University, Alexandria, Egypt; 2 Department of Pharmaceutical Microbiology and Immunology, Faculty of Pharmacy and Drug Manufacturing, Pharos University, Alexandria, Egypt

**Keywords:** Biofilm biomass, *S. aureus*, Subinhibitory concentration, qPCR, Spread plate technique

## Abstract

**Background:**

Biofilm formation is an essential virulence factor that creates a highly protected growth mode for *Staphylococcus aureus* (*S. aureus*) to survive in any hostile environment. Antibiotic sub-minimal inhibitory concentration (sub-MIC) may modulate the biofilm formation ability of bacterial pathogens, thereby affecting bacterial pathogenesis and infection outcomes. Intense antimicrobial therapy to treat biofilm-associated infections can control the pathogenic infection aggravation but cannot guarantee its complete eradication.

**Objective:**

This study aimed to assess the sub-MICs effect of 5 different antimicrobial classes on biofilm-forming capacity among *Staphylococcus aureus* clinical isolates using three different biofilm quantitation techniques.

**Methods:**

In this study, the effects of 5 different antimicrobial agents, namely, azithromycin, gentamicin, ciprofloxacin, doxycycline, and imipenem, at sub-MICs of 12.5%, 25%, and 50% were tested on 5 different clinical isolates of *S. aureus*. The biofilms formed in the absence and presence of different antimicrobial sub-MICs were then assessed using the following three different techniques: the crystal violet (CV) staining method, the quantitative PCR (qPCR) method, and the spread plate method (SPM).

**Results:**

Biofilm formation was significantly induced in 64% of the tested conditions using the CV technique. On the other hand, the qPCR quantifying the total bacterial count and the SPM quantifying the viable bacterial count showed significant induction only in 24% and 17.3%, respectively (Fig. 1). The difference between CV and the other techniques indicates an increase in biofilm biomass without an increase in bacterial growth. As expected, sub-MICs did not reduce the viable cell count, as shown by the SPM. The CV staining method revealed that sub-MICs of imipenem and ciprofloxacin had the highest significance rate (80%) showing an inductive effect on the biofilm development. On the other hand, doxycycline, azithromycin, and gentamicin displayed lower significance rates of 73%, 53%, and 47%, respectively.

**Conclusion:**

Exposure to sub-MIC doses of antimicrobial agents induces the biofilm-forming capacity of *S. aureus* via increasing the total biomass without significantly affecting the bacterial growth of viable count.

**Supplementary Information:**

The online version contains supplementary material available at 10.1186/s12879-024-09790-3.

## Introduction

Biofilm formation is an essential virulence factor in the pathogenesis of various opportunistic bacteria. Biofilm production is a natural process comprising both sessile and live cells that are characterized by their stable, irreversible attachment to a surface interface or each other. Compared with planktonic organisms, this sessile community of cells exhibits different phenotypic properties concerning their morphology, growth rate, and gene expression [1]. Biofilms are aggregates of microorganisms in the extracellular matrix (ECM) that firmly adhere to a surface. Generally, biofilms contain extracellular polymeric substances (EPSs), proteins, enzymes, lipids, nucleic acids and extracellular DNA (eDNA) [2].

Regarding the eDNA, there was a debate about the origin of the eDNA found, as it is present at the early stages of growth, which includes minimal cell lysis [3]. Davies et al. stated that sub-MICs may increase eDNA levels via a comparable nonlytic mechanism [4]. Biofilm eDNA can form a strong bond with motile cells within the biofilm as it accumulates in the areas where the biofilm has already formed. Moreover, it prevents existing motile cells from settling in biofilm-occupied habitats [5]. The cationic environment of eDNA and the biofilm complex structure may all limit the penetration of the charged antimicrobial agents [6].

Biofilms are protective barriers that allow bacteria to bypass the host immune response and confer tolerance to various antimicrobial agents, such as disinfectants and antibiotics. It is challenging to treat bacterial infections, especially those involving indwelling medical devices because biofilms allow the infection to persist [7]. *Staphylococci* are considered one of the most likely organisms that are responsible for hospital-acquired infections. Both coagulase-negative *Staphylococci* (CoNS) and *S. aureus* are implicated in intravenous catheter-related endogenous infections due to their ability to grow either on the catheter’s external surface or to reach an access port that allows their entrance [8].

Intense antibiotic therapy for biofilm-associated infections can reduce infection progression but cannot ensure total pathogen elimination. Away from pathogen eradication, the inconsistent use of antimicrobials while treating either device-related chronic bacterial infections like prosthetic valve endocarditis, or non-device-related ones as osteomyelitis and periodontitis, expose the included biofilm-forming bacteria to fluctuating Sub-MIC antimicrobial concentrations. Although the restricted penetration is considered a temporary mechanism, it grants the biofilm cells adequate time to accommodate more tolerance [9–11].

Sub-MIC can alter the biofilm matrix composition, leading to increased resistance and persistence [12]. Peptidoglycan inhibitors such as ampicillin and vancomycin at sub-MIC may induce biofilm formation, particularly that of eDNA-dependent biofilms, by triggering bacterial cell envelope stress. However, protein synthesis inhibitors such as erythromycin and kanamycin may inhibit biofilm formation. Another study revealed that the observed biofilm induction upon exposure to sub-MIC of cell wall synthesis inhibitor classes, not with the other antibiotic classes, including protein, DNA, folic acid, and RNA synthesis inhibitors [13, 14].

Multidrug resistance has increased worldwide, posing a significant public health threat. Several recent investigations have reported the emergence of multidrug-resistant bacterial pathogens from various origins, highlighting the need for the proper use of antibiotics. Additionally, routine application of antimicrobial susceptibility testing is essential to identify the appropriate antibiotics and screen for emerging MDR strains [15, 16]. Many studies demonstrated that the antimicrobial low concentrations can be considered as an inducer to the biofilm formation and development of bacterial resistance.

Bacteria within biofilms are usually exposed to sub-MIC doses of antimicrobial agents as the biofilm structure develops a concentration gradient that varies from planktonic cells to biofilm inner cells [17, 18]. The stimulatory or inhibitory effects of exposure to sub-MIC doses on biofilm formation remain controversial [19, 20]. The variability in the results was affected by the type of bacteria, antibiotics tested, and technique used to evaluate biofilm formation.

Different techniques have been developed for the quantification of biofilm formation, of which the “gold standard” is the crystal violet staining technique. This method was first described by O’Toole and Kolter in 1998 [21]. It is a low-cost assay that can be routinely performed easily and is appropriate for both qualitative and quantitative evaluation. It can measure the total biomass of biofilms because it stains both live and dead cells as well as other components of the biofilm matrix [22]. Despite its widespread use, CV has some limitations, making it difficult to distinguish between live and dead bacterial populations [23–25].

Quantitative real-time PCR (qPCR) can also be used to quantify biofilm-involved bacteria to a standard curve made of serially diluted bacterial suspensions. This method, however, can overestimate the count of biofilm bacterial cells due to the presence of free eDNA in the biofilm matrix [26] and other DNA produced from dead cells. With this method, both live and dead cells can be quantified [27, 28].

Determining the precise number of viable cells in the biofilm by the spread plate method may be challenging due to the various metabolic states of the cells, and the count may be underestimated due to the growth of bacterial cells in aggregates [25, 29]. Therefore, this study aimed to evaluate changes in biofilm formation in response to sub-MIC doses of antimicrobial agents using these three different techniques and to assess whether the effect is related to the extracellular material or biofilm-involved cells.

## Materials and methods

### Bacterial isolates and MICs of antimicrobial agents

This study included 5 clinical isolates of *S. aureus* from clinical specimens submitted to the microbiology laboratory of the Medical Research Institute (MRI), Alexandria University. Three of them were methicillin-susceptible (MSSA), and 2 were methicillin-resistant (MRSA) as shown in Table S1. *S. aureus* colonies were tested phenotypically for methicillin resistance by the Kirby-Bauer method using a cefoxitin disc (30 ug). Only cefoxitin-resistant isolates (≤ 21 mm) after 16–18 h were identified as MRSA. A Reference strain (ATCC 25923) was used as a methodology control in biofilm formation to confirm the CV staining method methodology in detecting the effect of sub-MIC on biofilm formation capacity (Figure S1).

The MICs of 5 different classes of antimicrobial agents, ciprofloxacin (fluoroquinolones class, EUROPEAN EGYPTIAN PHARM. IND., Cairo, Egypt), gentamicin (aminoglycosides class, Alexandria Co. for pharmaceutical & clinical industries, Cairo, Egypt), doxycycline (tetracycline class, El-Nile Co. for pharmaceutical & clinical industries, Cairo, Egypt), azithromycin (macrolide class, AUG pharma, Cairo, Egypt), and imipenem (carbapenem class, Sigma-Aldrich, UK), was determined according to the Clinical and Laboratory Standards Institute (CLSI; 2021) guidelines for broth microdilution susceptibility testing [30]. Briefly, bacterial isolates were cultivated on blood agar. After overnight incubation at 37 °C, 3–4 colonies were selected and emulsified in Müller Hinton broth (MHB) to obtain a turbidity of 0.5 McFarland. The bacterial suspension was diluted to a final bacterial count of 5 × 10^**5**^ CFU/mL and added to serial dilutions of the tested antibiotic ranging from 0.06 to 128 µg/mL in a total volume of 100 µL.

### Biofilm formation

Half McFarland standard was prepared from each isolate separately in MHB supplemented with 1% glucose, followed by 100-fold dilution. 50 µL of the bacterial suspension was mixed with 50 µL of sub-MIC concentrations of each antibiotic in a 96-well flat-bottomed sterile polystyrene microtiter plate. Wells containing MHB supplemented with glucose were used as a sterility control and for blanking purposes. Bacteria were incubated without disturbance for 24 h at 37 °C to allow biofilm formation. Each experiment was repeated at least 3 times. Each isolate was exposed to 3 different sub-MIC levels (12.5%, 25%, and 50%) of each of the 5 tested antimicrobial agents, indicating 15 different testing conditions for each antimicrobial.

### Evaluation of biofilm formation

#### Detection of total biomass by CV staining

After incubation, the contents of the wells were decanted and the plate was washed three times with 200 µL of sterile phosphate-buffered saline (PBS) by careful pipetting followed by simple tapping on a paper towel to remove any remaining nonadherent bacteria [31, 32].

The attached bacteria were heat-fixed at 60 °C for 60 min [32, 33]. The plates were then stained with 100 µL of 0.1% (w/v) CV solution and incubated at room temperature for 15 min to allow the stain to penetrate through the biofilm. The stain was removed, and the plate was washed several times with PBS and allowed to air dry. The dry-bound stain was resolubilized by adding 100 µL of absolute ethanol per well at room temperature for at least 30 min, after which the plates were kept with a lid [23, 24].

The absorbance at 620 nm was measured using a microplate reader (MTP reader, Biotek, VT, United States). The mean OD of the blank wells was subtracted from the OD of all wells. The OD in the presence of sub-MICs was then normalized relative to the mean OD in the absence of antibiotics (Figure S2, Figure S3).

#### Quantitation of biofilm DNA using qPCR

Initially, the biofilm was grown and washed as previously described, then, each well was scraped and resuspended in 100 µL of normal saline, transferred to a separate Eppendorf tube, and vigorously vortexed for 30 min, subjected to boiling at 100 °C in a water bath for 10 min, cooled on ice, and centrifuged at 15,000 g for 10 s [34]. The bacterial suspension was diluted 100-fold in sterile distilled water and used as a template for qPCR. In parallel, to create a standard curve, serial 10-fold dilutions (from 10^8^ to 10^2^ CFU/mL) of each of the tested bacterial isolates were prepared and subjected to qPCR.

Each qPCR consisted of 7.5 µL of Promega GoTaq^®^ qPCR Master mix (Promega, CITY), 0.3 µM forward (5’ ACTCCTACGGGAGGCAGCAGT 3’), and reverse primers (5’ TATTACCGCGGCTGCTGGC 3’) targeting the 16 S rRNA gene and 2 µL of diluted bacterial suspension. The qPCR amplification was accomplished using a Stratagene MX3000P (Agilent Technologies, USA). The thermal cycling was adjusted as described in the Master Mix protocol, with an annealing temperature of 60 °C [35].

The standard curve was subsequently used to determine the relative amount of DNA in each biofilm-formation experiment. linear regression was used to calculate the slope (S) of the standard curves. To obtain the total bacterial count, the test threshold cycle (CT) from the melting curves was compared to the created standard curve. The mean quantitative value for each triplicate sample was determined and subsequently normalized relative to the baseline value in the absence of antibiotics (Figure S4, Figure S5).

#### Detection of viable cells using the spread plate method

To evaluate the count of the viable cells within the biofilm for *S. aureus* strains, the biofilm was scraped and resuspended as previously described, the bacterial suspension was centrifuged for 10 min at 10,000 rpm, and the pelleted cells were resuspended in 100 µL of PBS with vigorous vertexing followed by five successive 10-fold dilutions. Using a Drigalski spatel, the whole contents of the Eppendorf tube containing diluted bacteria were inoculated onto mannitol salt agar plates using the spread plate method. The plates were incubated at 37 °C for 24 h. Only plates showing colony numbers between 25 and 250 colonies were counted visually, and the remaining plates were discarded. The test was performed in triplicate, and the mean value of the counted viable biofilm cells was calculated and then normalized to the baseline count in the absence of antimicrobial drugs [29, 36, 37].

#### Statistical analysis

All datasets were created in spreadsheets by Microsoft Excel software and uploaded to RStudio (version 2022.02.0 + 443) as csv files for both descriptive and statistical analysis via the R programming language. Then, the normality of each dataset was assessed statistically by the Shapiro‒Wilk test. If the data did not fulfill the parametric assumptions, the Friedman test was used, followed by the Dunn test for post hoc pairwise comparisons. The chi-square test was used to compare categorical variables. Differences between each sub-MIC category (12.5%, 25%, and 50%) of each antibiotic and its baseline (no antibiotics exposure) for each isolate of S. *aureus* were assessed by paired t-tests to determine the significance of each sub-MIC effect on each isolate. After the determination of significant sub-MIC categories for each isolate, a qualitative analysis of overall categories of not significant, significant decrease, and a significant increase was performed using the chi-square test goodness of fit to determine the direction of the overall effect on biofilm formation determined by each method on the tested *Staphylococcus* spp.

## Results

### Biofilm total biomass detection by CV staining

The CV biofilm quantitation method showed that biofilm formation was significantly induced in 64% of the tested conditions relative to the control. Exposure to sub-MIC doses of both imipenem and ciprofloxacin had the highest significance rate (80%), followed by doxycycline (73%), azithromycin (53%) and gentamicin (47%). (*p* value = 0.024). (Table 1; Fig. 2, Table S2)

**Table 1:** Comparison of the three biofilm evaluation methods’ significant results

Total tested conditions = 15 (100%)	Total significance rate of different Sub-MIC on *S. aureus* clinical isolate biofilm formation capacity by different methods used.					
	CV		qPCR		Spread plate	
	Induction (64%)	Inhibition (2.6%)	Induction (24%)	Inhibition (14.6%)	Induction (17.3%)	Inhibition (1.3%)
Azithromycin	53%		0%		27%	
	8	0	0	0	4	0
Ciprofloxacin	80%*		67%		6.7%	
	12*	0	7	3	1	0
Doxycycline	73%*		20%		6.7%	
	9*	2	2	1	1	0
Gentamicin	47%		6.7%		27%	
	7	0	1	0	3	1
Imipenem	80%*		73%		27%	
	12*	0	8*	3*	4	0

The asterisks indicate the Total significance level (*p value < 0.05) considering all the isolates showed a significant effect statistically. Isolates showed no change in their response are not illustrated. Total effects for all tested conditions of the 5 clinical isolates with the 3 Sub-MIC of each antibiotic are presented as percentages

**Fig. 1:**
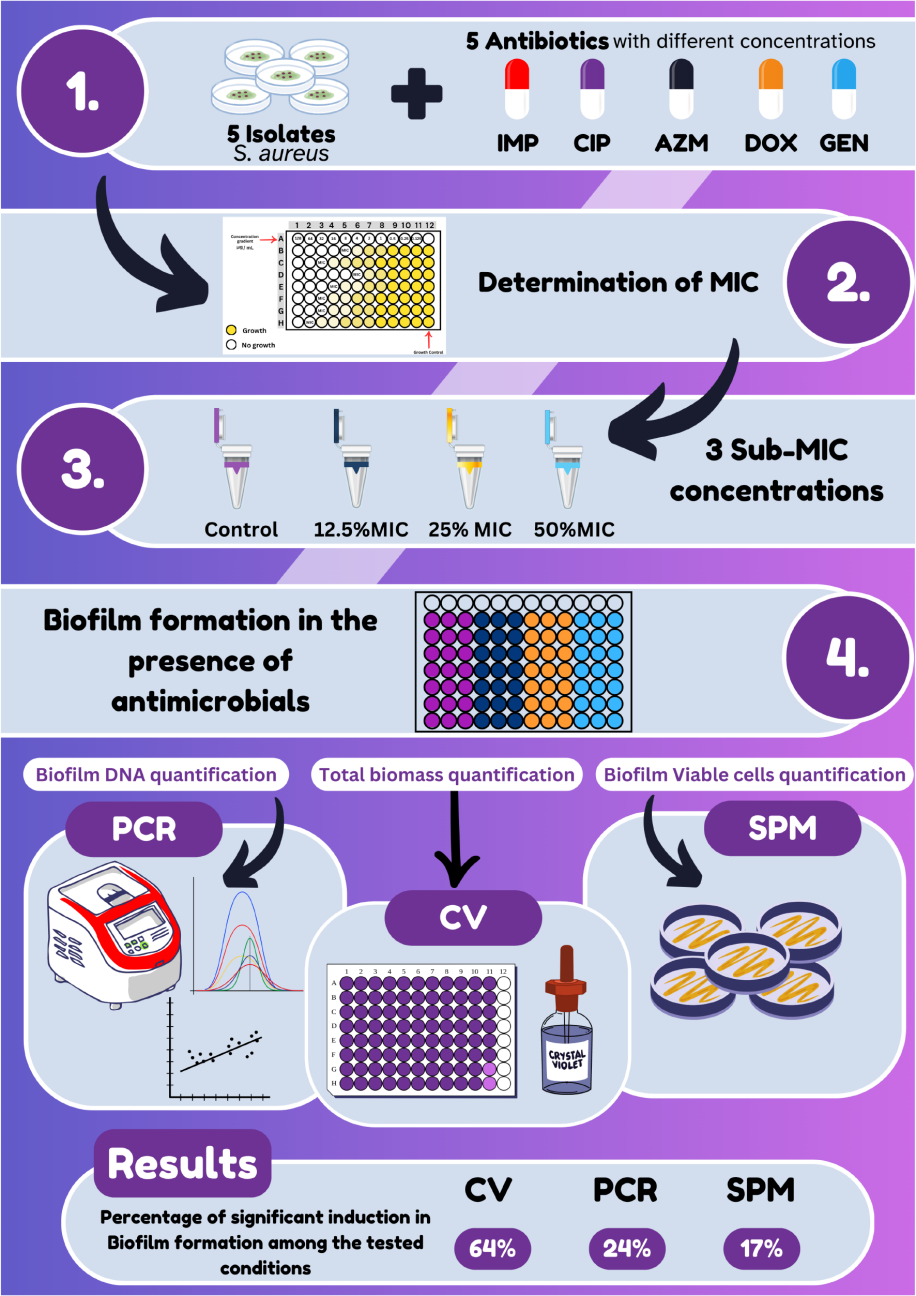
Graphical representation of the study abstract. IMP; imipenem, CIP; ciprofloxacin, AZM; azithromycin, Dox; doxycycline, GEN; gentamicin, CV; crystal violet, SPM; Spread plate method, PCR; Polymerase chain reaction, MIC; Minimum inhibitory concentration

**Fig. 2:**
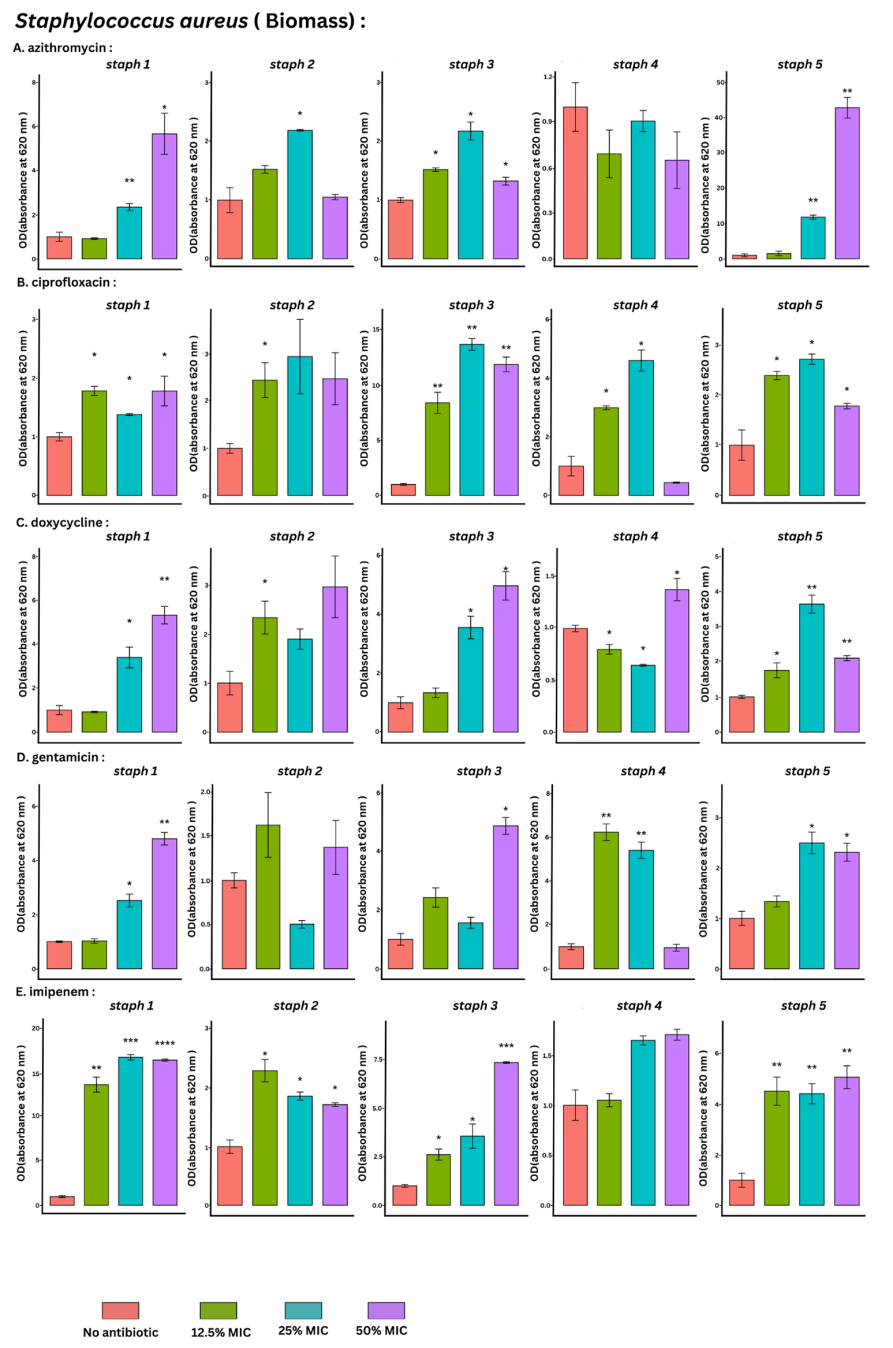
Bar plots with error bars (mean ± SD) of optical density (OD) with absorbance at 620 nm of *S. aureus* isolates (*n* = 5) by CV staining method at baseline (no antimicrobial) and at 3 different sub-MICs (12.5%, 25% and 50%) of azithromycin, ciprofloxacin, doxycycline, gentamicin and imipenem, respectively. All data were normalized to their mean baseline (no antimicrobial). *Asterisks indicate the statistical significance obtained by paired t- test. The number of asterisks indicates the significance level (*p value < 0.05, **p value < 0.005, ***p value < 0.0005)

All antimicrobial agents produced only a significant biofilm induction except for doxycycline which significantly induced biofilm formation in 9/15 (60%) and significantly reduced biofilm formation in 2/15 (13%) of the tested conditions.

At the sub-MIC level, no association could be predicted between the sub-MIC level and the biofilm formation observed changes as shown for ciprofloxacin, azithromycin, doxycycline, and gentamycin. All tested sub-MIC levels showed the same significance rate for imipenem.

A Reference strain (ATCC 25923) was involved and the results obtained are shown in (Figure S1) confirming the used methodology. Exposure to sub-MIC doses of both imipenem and ciprofloxacin showed a significant biofilm formation induction.

### Total bacterial count by qPCR method

Biofilm growth was significantly induced in 24% of the tested conditions relative to the control without antibiotic exposure. Imipenem had the highest significance rate (73%), followed by ciprofloxacin (67%), doxycycline (20%), and gentamicin (6.7%). Azithromycin showed neither a stimulatory nor an inhibitory effect, and the results showed a statistically nonsignificant effect. (*p-value* = 0.0001075)

Both imipenem and ciprofloxacin significantly induced biofilm biomass bacterial count in 8/15 (53%) and 7/15 (46%) of the tested conditions. On the other hand, gentamicin and doxycycline showed the least biofilm induction among the isolates, at 1/15 (6.67%) and 2/15 (13.33%), respectively.

**Fig. 3:**
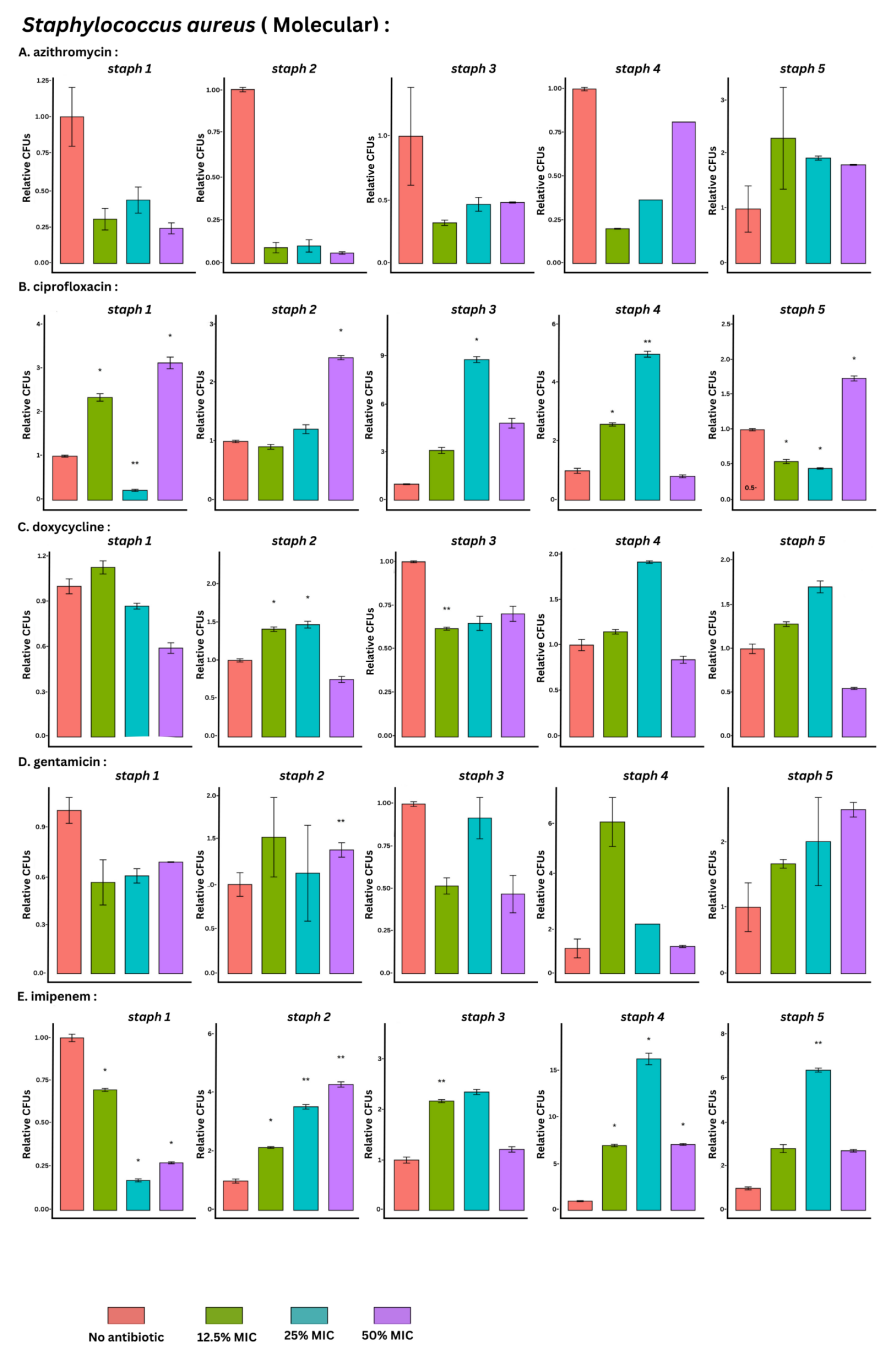
Bar plots with error bars (mean ± SD) of CFU count of *S. aureus* isolates (*n* = 5) by qPCR method at baseline (no antimicrobial) and at 3 different sub-MICs (12.5%, 25% and 50%) of azithromycin, ciprofloxacin, doxycycline, gentamicin and imipenem, respectively. All data were normalized to their mean baseline (no-antimicrobial).*Asterisks indicate the statistical significance obtained by paired t- test. The number of asterisks indicates the significance level (*p value < 0.05, **p value < 0.005, ***p value < 0.0005)

Among the other antimicrobial agents tested, imipenem and ciprofloxacin had the most significant rate of biofilm induction at all three tested sub-MIC (Table 1; Fig. 3, Table S3).

### Viable cell count by the SPM

**Fig. 4:**
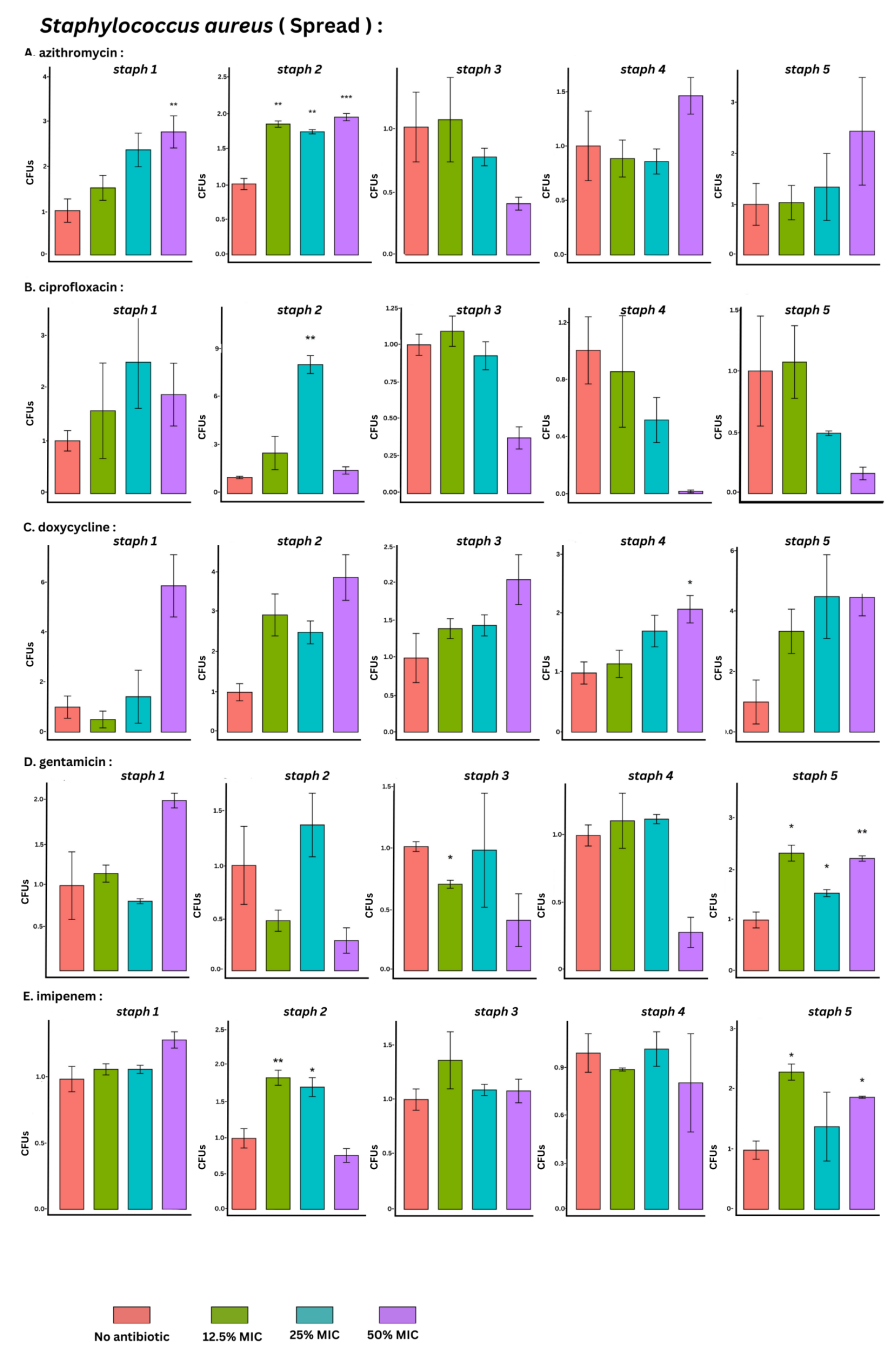
Bar plots with error bars (mean ± SD) of viable cell count of *S. aureus* isolates (*n* = 5) by spread plate method at baseline (no antimicrobial) and at 3 different sub-MICs (12.5%, 25% and 50%) of azithromycin, ciprofloxacin, doxycycline, gentamicin and imipenem, respectively. All data were normalized to their mean baseline (no antimicrobial). *Asterisks indicate the statistical significance obtained by paired t- test. The number of asterisks indicates the significance level (*p value < 0.05, **p value < 0.005, ***p value < 0.0005)

The spread plate method targeting viable bacterial count showed significant induction only in 17.3% of the tested conditions. Among the tested antibiotics tested, imipenem, azithromycin and gentamicin sub-MICs showed the highest significance rate (27%). azithromycin and imipenem significantly induced the viable cell count in 4/15 (27%) of tested conditions, while gentamicin induced the viable cell count in 3/15 (20%) of the tested conditions. A smaller colony variant (SCVs) of *S. aureus* was observed while counting (Table 1; Fig. 4, Table S4).

### Comparative evaluation of the significant results

Regarding the three employed methods, the significance rate of the data was greater for the CV staining approach (66.6%) than for the qPCR and spread methods (38.6% and 18.6%, respectively) (Table 1). There was a greater percentage of significant induction in 64% of the investigated conditions, compared to 24% with qPCR and 17.3% with SPM. The CV significance rate of the obtained results exceeded those of the other two approaches, exhibiting a much greater induction than inhibition rate, especially for the imipenem and ciprofloxacin-tested conditions.

Compared with the other two methods, the CV staining method obtained the highest significance rate (80%) for imipenem, which was 73% and 27% for qPCR and SPM, respectively. The induction effect was mostly recognized in the CV staining method in 80% (12/15) of the tested conditions, followed by the qPCR method in 53% (8/15) of the tested conditions and the least induction with SPM in 20% (3/15). The CV staining method and SPM showed only a significant induction in biofilm formation.

The CV staining method and qPCR methods demonstrated a greater significance rate with ciprofloxacin (80% and 67%, respectively) than with SPM (6.7%). CV staining method showed more prominent biofilm induction in 80% (12/15) of the tested conditions, compared with those of the other two methods. The qPCR method was the only method that showed that both imipenem- and ciprofloxacin-triggered inhibitory effects on 20% (3/15) of the tested conditions.

CV staining revealed that doxycycline results had the highest significance rate in (73%) of tested conditions regarding the other two methods, with induction in 60% of them (9/15). Both the CV method and qPCR revealed the inhibitory effect among the significant results on 2/15 and 1/15 of the tested conditions, respectively. while SPM showed no observed inhibitory effect.

The CV method results revealed that azithromycin tested conditions displayed a much higher significance rate in 8/15 (53%) than in SPM in 4/15 (27%). Induction was the only effect detected with the CV and SPM.

CV staining revealed that the lowest significance rate and induction effect were detected for gentamicin at sub-MICs in 7/15 (47%) of the tested conditions. Regarding the other two methods, an inhibitory effect was detected by the SPM with gentamicin.

Biofilm inhibition was obvious with qPCR with three antimicrobial agents, ciprofloxacin, imipenem, and doxycycline, at sub-MIC concentrations. A higher inhibition rate was detected with qPCR in 14.6% of the tested conditions, while the lowest inhibition rate was detected with SPM in 1.3% of the significant results (Table S2, Table S3, Table S4).

## Discussion

In this study, we evaluated the biofilm formation of five different *S. aureus* clinical isolates in response to five different antimicrobial agents—azithromycin, gentamicin, ciprofloxacin, doxycycline, and imipenem—at three different sub-MIC levels (12.5%, 25%, and 50%) using three distinct techniques. Several techniques are used for biofilm evaluation, and we employed 3 techniques in this study to detect the bacterial response and measure the biofilm formation capacity after exposure to different sub-MICs, as investigated by other researchers [38, 39]. The difference between the 3 methods can be attributed to the fact that the CV staining method can measure the whole biofilm biomass, while the other two methods, comprising the qPCR method, a molecular technique that counts the biofilm-involved cells in relation to the nucleic acid present, and the SPM, a colony counting method, can only measure the biofilm viable cell count. The fact that the CV staining method stains the whole biofilm biomass, including live and nonviable cells as well as polymeric matrix components, can explain its ability to detect most of the changes in biofilm formation capacity [40–42].

In this study, more than one method was employed to observe changes in total biofilm biomass and the effect of sub-MIC on the involved viable and dead cells. The CV staining method had the highest significance rate detected with 66.6% of the strains under the tested conditions, compared with 38.6% with the qPCR method and 18.6% with the other methods. This significance rate showed biofilm induction in 64% of the tested conditions compared to 24% and 17.3% in the qPCR and SPM, respectively. The observed induction showed that sub-MIC doses of antimicrobial agents are more effective at increasing the total biofilm biomass, including that of the extracellular matrix (ECM), than are the corresponding involved cell counts.

Regarding biofilm quantification, multiple approaches allow biofilm quantification and the observation of changes. Some are conventional, such as CV staining and colony forming unit (CFU) counting methods, whereas others are more recent, such as spectroscopic assays and scanning electron microscopy imaging. The biofilm can be measured directly by flow cytometry, microscopic cell counting, confocal laser scanning microscopy (CLSM), and fluorescence microscopy or indirectly through microtiter plate assays. Fluorescence microscopy can help characterize biofilms clearly by detecting the light emitted after the incorporation and absorption of fluorescent dyes, such as SYTO and propidium iodide dyes, within the biofilm structure. However, not all strains can successfully penetrate the biofilm complex structure and maintain biofilm viability [37, 43]. Moreover, changes in biofilm biomass could be due to the presence of ECM-related components; therefore, quantifying biofilm-related components, such as ECM, RNA, DNA, proteins, and carbohydrates, can aid in biofilm characterization. Although multiple approaches can be applied, the extraction and purification of the biofilm components can be challenging. Therefore, to select a suitable technique for biofilm assessment, one should consider its applicability and reliability.

The CV staining method is used to detect total biofilm biomass changes and is considered an affordable and simple method for detecting most if not all, biofilm-forming bacteria. This approach of direct biofilm detection and indirect total biomass quantification provides observable data that reflect the biofilm formation capacity: the more biofilm formed; the more CV stain was absorbed. Although multiple procedures and repetitions are involved in obtaining reproducible findings, many samples can be analyzed in parallel [25]. Haney et al. observed little discrepancy among the replicates; however, others found obvious differences. The variation may be attributed to the medium composition and the dead-bound bacteria, which can affect the accuracy, particularly in the presence of antimicrobial agents [44–46]. The growth conditions, bacterial strains, and reader used may influence the measurement of the absolute values. Consequently, a control must be employed for normalization to minimize variability and enable proper comparison between duplicates [47]. Throughout the investigation, we maintained the same conditions for *S. aureus*, used MHB as a growth medium during biofilm formation, and measured the optical density (OD) using the same reader to reduce as much variation as possible between the replicates. Nevertheless, wells containing only growth media were used for sterility control and blanking purposes. Triplicate mean values were calculated to compare the OD values of each tested condition to the values measured in the absence of antibiotics.

We also employed a qPCR molecular approach, which is considered a reliable rapid technique, to assess the changes in the total number of biofilm bacterial cells in the presence and absence of sub-MIC. In our study, the qPCR results showed a 38.6% significance rate under the tested conditions. Compared with the CV staining technique, there was lower biofilm induction and more biofilm inhibition in 24% and 14.6% of the tested conditions, respectively. This might suggest that rather than impacting the total biomass, sub-MIC can have an inhibitory effect on biofilm-involved cells. A study revealed that there is a good correlation between CFU counts and qPCR quantification methods, as long as the bacterial viability in biofilms is relatively high [48]. However, qPCR showed a limited ability to distinguish between viable and dead cells. Additionally, a common drawback is cell number overestimation due to the presence of other sources of nucleic acid, such as eDNA [28]. This method can show the inhibitory and induction effects of sub-MICs but cannot determine whether the effect is due to live or dead bacterial cells. Therefore, we employed another method to count the number of biofilm-associated cells.

To measure biofilm viability, the SPM was used rather than other recently used approaches, such as the use of tetrazolium salts (XTTs), allowing viability assessment via microscopy or spectroscopy [37]. The results of SPM showed a significance rate in 18.6% of the results showing an increase in the biofilm viable count of 17.3% of the tested conditions and an inhibition of 1.3%. The low inhibitory effect detected among the viable cell counts in our study was expected due to bacterial cell survival at concentrations sub-MIC of the antimicrobial agents. Despite not being statistically significant, there was an increase in the biofilm viable cell count similar to that detected by Wojnicz and Tichaczek-Goska, who noticed more live bacteria in the presence of sub-MIC antibiotics than in the controls after biofilm development over 24- and 48-hour periods [49].

The traditional SPM quantifies biofilm viable cells without the need for specialized equipment. In practice, this method is tedious and time-consuming. To ensure consistency and reproducibility, triplicates are carried out, which may take days to prepare, requiring a good plating technique and medium preparation. Sometimes it may be difficult to obtain a significant colony number, as in the case of minimal biofilm formation [50]. Accurate determination of colony number may be difficult due to bacterial clumping, the different metabolic states of the bacterial cells, and human bias in manual counting. The colony counter can overcome these errors [25].

The three implemented techniques demonstrated that there was an increase in the total biofilm biomass revealed by the CV staining method. However, the inhibitory effect was detected by qPCR and SPM showing an effect on the biofilm-associated cells rather than its involved viable cells.

Chen et al. showed that the bacterial biofilm formation capacity can differ depending on the tested strain, related biofilm stage, and antimicrobial class. The presence of exopolysaccharides and eDNA in biofilms can hinder the diffusion of antimicrobial agents, allowing sensitive bacteria to survive [19]. Many studies have shown that sub-MIC per se can trigger biofilm growth [51, 52] and encourage bacterial aggregation [53]. Therefore, special attention is given to biofilm-forming bacteria as they are usually exposed to Sub-MIC doses of antimicrobials. In turn, biofilm formation may be induced instead of being eradicated due to enhancing the tolerance of sessile cells against the antimicrobial agents and increasing the mutagenesis selection within the biofilm [54].

Four mechanisms exist in biofilms that limit their sensitivity to antimicrobials involving the physical tolerance due to the restricted penetration, stress response of bacterial cells that can lead to overproduction in exopolysaccharides or one of the biofilm-involved components, the altered microenvironment, and mainly the persister cells which are displaying dormancy so they can survive against the cell growth targeting antimicrobials and against the stress conditions [55].

In this study, we recognized that the effect triggered by each antimicrobial agent varied among the antimicrobial class and the tested conditions. The alterations exerted were mostly associated with the biofilm total biomass rather than its involved cells. The antimicrobial drugs that exhibited the highest significant rate were imipenem and ciprofloxacin, especially according to the qPCR and CV techniques. According to the three employed methods, the significance rates were higher in CV and qPCR than in SPM for imipenem and ciprofloxacin. The significance rates for imipenem were 80%, 73%, and 28%, and for ciprofloxacin were 80%, 64%, and 6.7%, respectively. Subsequently, doxycycline showed a significance rate of 73%, 20%, and 6.7% in the CV, qPCR and SPM methods, respectively. The azithromycin significance rate among the tested conditions was found to be only in the CV method and SPM, while it was much higher in the CV method (53%) than in SPM (27%). This finding indicates that the use of azithromycin as a bacteriostatic trigger changes the bacterial biofilm ECM and viable cell count without affecting the involved nonviable cells. However, among the antimicrobial agents tested, gentamicin had the lowest significance rate. gentamicin with its tested conditions showed a significance rate of 47%, 27%, and 6.7% by CV, qPCR, and the SPM, respectively. Therefore, gentamicin is the least antimicrobial agent that affects the among the antimicrobial agents tested; gentamicin had the lowest significance rate. Therefore, gentamicin is the antimicrobial agent with the least effect on *S. aureus* biofilm formation capacity.

Regarding cell wall inhibitors, we tested bacterial biofilm formation in the absence and presence of imipenem. There was an induction in biofilm formation in 80% (12/15) and 53% (8/15) of the tested isolates according to CV and qPCR, respectively. Ng et al. reported that clinical and community-associated MRSA strains exhibited greater bacterial adhesion upon exposure to β-lactam sub-MICs [56]. Another study conducted by Haddadin et al. demonstrated a trigger for *S. aureus* biofilm formation at various sub-MICs (2.81-45%) [57]. Although we observed biofilm formation by CV, inhibition in the total cell count by qPCR was observed in only 20% (3/15) of the strains tested, indicating that the imipenem inhibitory effect can be due to the involved biofilm nucleic acid and not the bacterial adhesion.

Sub-MIC of antimicrobial agents affects bacterial response and biofilm formation capacity through different reported mechanisms [17, 58]. A previous study revealed that the sub-MIC of ampicillin increased biofilm formation in *Staphylococcus intermedius* biofilms via the autoinducer-2/LuxS signaling pathway [59]. Other researchers have reported that cephalexin can promote cell adhesion by enhancing the hydrophobicity of the *S. aureus* cell surface [60]. Further investigations reported that β-lactam sub**-**MICs increase the expression of *S. aureus* exotoxins and adhesion factors [61, 62].

Kaplan et al. attributed the same observation to the induction of bacterial eDNA and the promotion of auto-aggregation [63]. An increase in eDNA was reported in many studies in which exposure to sub-MIC concentrations of vancomycin promoted biofilm formation in *S. epidermidis* [64] and *S. aureus* [65, 66]. Many researchers have reported increased endotracheal tube (ETT)biofilm development, infection severity, and mucus production. These studies revealed a correlation between systemic vancomycin sub-therapeutically administered concentrations and an increase in biofilm thickness [67] and the occupied area in vivo [68].

Regarding DNA synthesis inhibitors, we tested bacterial biofilm formation in the absence and presence of ciprofloxacin. Ciprofloxacin showed a greater significance rate with the CV method (80%) and with the qPCR method (67%) than with the SPM method (6.7%). The induction rate was highest with the CV staining method, followed by qPCR in 80% (12/15) and 46% (7/15) of the tested conditions, respectively, but lowest in the SPM. Sometimes the biofilm complex structure is easily penetrated by ciprofloxacin, but in other situations, the overproduction of polysaccharides not only prevents antibiotic penetration but also alters the physiological state of the aggregated bacteria, reducing their ability to absorb nutrients and oxygen [54]. The SOS response, which can be triggered by genotoxic stressors such as UV light, can also be triggered by sub-MIC fluoroquinolone antimicrobial agents such as ciprofloxacin, which was found to induce explosive cell lysis [69]. Therefore, a change in biofilm formation capacity can be detected using CV and qPCR but not with SPM, which can result in false inhibition.

The increase in biofilm density detected by CV staining has also been reported in other studies. ciprofloxacin promotes *S. aureus* bacterial adhesion by upregulating the expression of proteins that bind to fibronectin, which in turn enhances bacterial adhesion to host tissue [17]. Rosman et al. reported that the polysaccharide intercellular adhesin of *S. aureus*, which is the primary component of the biofilm ECM, is produced by *ica* operon enzymes, and certain antimicrobial agents at sub-MIC levels may impact its expression [42].

As with the previously discussed antimicrobial agents, doxycycline and azithromycin biofilm induction effect was more observed with the CV staining method. Doxycycline showed an induction in 9/15 (60%) of the tested isolates, whereas azithromycin showed an induction in 8/15 (53%).

Compared with those of the other antimicrobial agents, the sub-MIC of gentamicin had the lowest significance rate (47%), and the lowest induction rate was detected in 7/15 of the tested conditions with the CV staining method. There was an inducing effect observed with the other two methods but to a lesser extent than that detected with the CV method. An inhibitory effect was also detected with SPM in only one isolate. Schaible et al. indicated that an anaerobic biofilm environment may allow the development of oxygen tension and induce changes in the expression of energy metabolism genes, affecting aminoglycoside functioning [70]Also, the complex biofilm structure due to the presence of the negatively charged polysaccharides makes it difficult for large molecules and positively charged antibiotics such as aminoglycosides to penetrate allowing the buildup of sub-MIC within the biofilm [36]. However, in other cases, antimicrobial agents such as quinolones and uncharged beta-lactams may be more easily accessible [37].

The sub-MIC of each antimicrobial may result in two different effects (either stimulatory or inhibitory potential) on biofilm formation on different bacterial isolates of the same species. Our findings were similar to a study by Yousefpour et al., who observed that his clinical isolates responded differently to sub-MIC doses of gentamicin except he was investigating the effect on *P. aeruginosa*. He stated that the gentamicin effects on biofilm development were stimulatory at a ^**1**^**/**_**2**_ MIC and inhibitory at a ^**1**^**/**_**4**_ MIC in some isolates; however, other isolates responded differently. A ^**1**^**/**_**2**_ MIC of gentamicin promoted biofilm development in several isolates, while a ^**1**^**/**_**4**_ MIC had no impact and vice versa [20].

The inhibitory effect was obvious in qPCR for three antimicrobial agents, ciprofloxacin, imipenem, and doxycycline, at their sub-MIC. Both imipenem and ciprofloxacin had inhibitory effects on 20% (3/15) of the tested conditions according to qPCR. However, the qPCR technique confirmed that the inhibitory effect of imipenem was not related to the viable cell count of the biofilm, as no inhibition was detected in the viable cell count by SPM. A study by Jo and Ahn et al. showed that β-lactams may inhibit the adhesion of the tested *S. aureus* bacteria. In addition, 3080 CFU of *S. aureus* CCARM were reduced in the presence of levofloxacin [71]. For the other two methods, the inhibitory effect of doxycycline was detected via CV staining, and that of gentamicin was detected via SPM.

Among the tested conditions, doxycycline was the only antimicrobial agent that inhibited biofilm formation according to CV staining. We found minimal biofilm inhibition with only 2/15 and 1/15 of the tested conditions by the CV method and qPCR, respectively. Similarly, a study revealed that low concentrations of tigecycline reduced biofilm formation even when planktonic cell growth was not significantly affected [72]. A reduction in biofilm formation upon exposure to some antimicrobial classes at sub-MIC was reported by Zheng et al., who reported that ^**1**^**/**_**4**_ sub-MIC of azithromycin, clindamycin, vancomycin, or daptomycin can significantly prevent the formation of *S. aureus* biofilms [73]. Another study showed that amikacin can reduce the ability of bacteria to produce biofilms [49].

There is a debate regarding the relationship between bacterial sensitivity to antimicrobial agents and biofilm formation capacity. Yu et al. reported an inverse correlation between the degree of biofilm formation and bacterial methicillin susceptibility [74]. In this study, CV staining revealed biofilm induction at 12.5% MIC of ciprofloxacin, with greater induction in MSSA (isolates 2, 3, and 4) than in MRSA (isolates 1 and 5). The degree of induction by azithromycin was greater among the MRSA isolates (isolates 1 and 5) (Fig. 2). Within MRSA and MSSA, there is a fundamental distinction. Although the development of biofilms in MSSA is *icaADBC* dependent and depends on PIA/PNAG adhesin proteins, biofilm formation in MRSA is *icaADBC* independent because of the availability of additional components and the environment, such as an acidic growth medium and glucose [75]. Bacitracin induced biofilm formation in both resistant and sensitive strains of *Streptococcus mutans* at ^**1**^**/**_**8**_ and **½** MIC, respectively, and promoted horizontal gene transfer within the eDNA-dependent biofilm [17, 58].

The limitations of this study were the small sample size, which we attempted to compensate for by using 3 different sub-MICs of each antimicrobial agent and 3 different biofilm evaluation techniques to obtain confirmed reproducible results and the use of only one representative antimicrobial agent for each of the 5 classes. Thus, to conduct further research, a large sample size and deeper examination of the mechanisms underlying changes in biofilm development by targeting the genes linked to QS and biofilm formation are needed. Nevertheless, it would be more important to test other classes of antimicrobial agents to further investigate the differences in the effects of bactericidal and bacteriostatic agents. Additionally, another recent biofilm evaluation technique can be added to the conventional methods used to confirm the obtained results.

## Conclusion

Different methods for evaluating biofilm formation have varying degrees of success in identifying how sub-MICs affect biofilm development. The three implemented techniques demonstrated that there was an increase in the biofilm biomass, as evidenced by the CV staining results, rather than in the biofilm cell count. However, the CV staining method approach overlooked the effect without determining growth and viability changes. According to the study’s findings, additional perspectives must be taken into account when treating biofilm-related infections. Exposure to sub-MIC doses of antimicrobial agents might be a stressor for biofilm-forming *S. aureus* that can induce its biofilm-forming potentiality. Therefore, the use of antibiotics might not only fail to eradicate the biofilm formed during infection but also worsen it.

## Electronic supplementary material

Below is the link to the electronic supplementary material.

Supplementary Material 1

## Data Availability

All data generated or analyzed during this study are included in this published article and its supplementary information file.
