# Correction: Antimicrobial Sub-MIC induces Staphylococcus aureus biofilm formation without affecting the bacterial count

**DOI:** 10.1186/s12879-024-10038-3

**Published:** 2024-10-22

**Authors:** Raghda Elawady, Aliaa G. Aboulela, Ahmed Gaballah, Abeer A. Ghazal, Ahmed N. Amer

**Affiliations:** 1 Department of Microbiology, Medical Research Institute, Alexandria University, Alexandria, Egypt; 2 Department of Pharmaceutical Microbiology and Immunology, Faculty of Pharmacy and Drug Manufacturing, Pharos University, Alexandria, Egypt

**Correction** **:** **BMC Infect Dis 24, 1065 (2024)**

**https://doi.org/10.1186/s12879-024-09790-3**

Following publication of the original article [1], we have been notified that Figure 2 was missing and a duplicate of Figure 3 was placed in its spot. We have now replaced originally published Figure 2 with the correct figure.

The original article has been corrected.

## References

[1] Elawady, et al. Antimicrobial Sub-MIC induces Staphylococcus aureus biofilm formation without affecting the bacterial count. BMC Infect Dis. 2024;24:1065. 10.1186/s12879-024-09790-3. 39342123 10.1186/s12879-024-09790-3 PMC11438285

