# Supplementary material for: Antimicrobial Sub-MIC induces Staphylococcus aureus biofilm formation without affecting the bacterial count
Source: BMC Infect Dis. 2024 Sep 28;24:1065. doi: 10.1186/s12879-024-09790-3 (PMC11438285; doi:10.1186/s12879-024-09790-3)

**Table S 1 A table including the strains' names, sources, collecting sites:**

| **strain** | **source** | **Collecting site** |
| --- | --- | --- |
| Staph1 | CRBSI | IV catheter |
| Staph2 | CRBSI | IV catheter |
| Staph3 | CRBSI | IV catheter |
| Staph4 | Cystic fibrosis | Sputum |
| staph5 | Cystic fibrosis | Sputum |

(CRBSIs); Central-venous-catheter-related bloodstream infections

**Table S2 Effect of sub-MICs of different antimicrobial agents on S. aureus biofilm formation capacity according to the crystal violet staining method**

| Isolate ID | azithromycin | ciprofloxacin | doxycycline | Gentamicin | Imipenem |
| --- | --- | --- | --- | --- | --- |
| *Staph 1* | 12.5% N.S.  25% sub-MIC; **  50% sub-MIC; * | 12.5% sub-MIC;  25% sub-MIC;  50% sub-MIC;  * | 12.5% N.S  25% sub-MIC; *  50% sub-MIC; ** | 12.5% N.S  25% sub-MIC;  *  50% sub-MIC;  ** | 12.5% sub-MIC;  **  25% sub-MIC;  ***  50% sub-MIC;  **** |
| *Staph 2* | 12.5% N.S  25% sub-MIC; *  50% N. S | 12.5% sub-MIC; *  25% N.S  50% N. S | 12.5% sub-MIC; *  25% N.S  50% N. S | N.S. | 12.5% sub-MIC;  25% sub-MIC;  50% sub-MIC;  * |
| *Staph 3* | 12.5% sub-MIC;  25% sub-MIC;  50% sub-MIC;  * | 12.5% sub-MIC;  25% sub-MIC;  50% sub-MIC;  ** | 12.5% N.S  25% sub-MIC; *  50% sub-MIC; * | 12.5% N.S  25% N.S  50% sub-MIC;  * | 12.5% sub-MIC; *  25% sub-MIC;  *  50% sub-MIC;  *** |
| *Staph 4* | N.S. | 12.5% sub-MIC;  25% sub-MIC;  *  50% N. S | 12.5% sub-MIC;  25% sub-MIC;  50% sub-MIC;  * | 12.5% sub-MIC;  25% sub-MIC;  **  50% N. S | N.S. |
| *Staph 5* | 12.5% N.S  25% sub-MIC; **  50% sub-MIC; ** | 12.5% sub-MIC; *  25% sub-MIC; *  50% sub-MIC; * | 12.5% sub-MIC; *  25% sub-MIC; **  50% sub-MIC; ** | 12.5% N.S  25% sub-MIC; *  50% sub-MIC; * | 12.5% sub-MIC;  25% sub-MIC;  50% sub-MIC;  ** |

Each experiment was performed in triplicate. Statistical analysis was performed by paired t tests. N.S. = not significant. The number of asterisks indicates the significance level (**p* value < 0.05, ***p* value <0.005, ****p* value <0.0005).

**Table S3 Effect of sub-MICs of different antimicrobial agents on the DNA content of S. aureus biofilms, as determined by qPCR.**

| Isolate ID | azithromycin | ciprofloxacin | doxycycline | Gentamicin | imipenem |
| --- | --- | --- | --- | --- | --- |
| *staph1* | N.S. | 12.5% sub-MIC  ***  25% sub-MIC  ****  50% sub-MIC  *** | N.S. | N.S. | 12.5% sub-MIC  25% sub-MIC  50% sub-MIC  *** |
| *staph2* | N.S. | 12.5% N.S.  25% N.S.  50% sub-MIC  *** | 12.5% sub-MIC  25% sub-MIC  ***  50% N.S. | 12.5% N.S.  25% N.S.  50% sub-MIC  **** | 12.5% sub-MIC  ***  25% sub-MIC  50% sub-MIC  **** |
| *staph3* | N.S. | 12.5%  25% sub-MIC  ***  50% N.S. | 12.5% sub-MIC  ****  25% N.S.  50% N.S. | N.S. | 12.5% sub-MIC  ***  25% N.S.  50% N.S. |
| *staph4* | N.S. | 12.5% sub-MIC  ***  25% sub-MIC  ****  50% N.S. | N.S. | N.S. | 12.5% sub-MIC  25% sub-MIC  50% sub-MIC  *** |
| *staph5* | N.S. | 12.5% sub-MIC  25% sub-MIC  50% sub-MIC  *** | N.S. | N.S. | 12.5% N.S.  25% sub-MIC  ****  50% N.S. |

Each experiment was performed in triplicate. Statistical analysis was performed by paired t tests. N.S. = not significant. The number of asterisks indicates the significance level (**p* value < 0.05, ***p* value <0.005, ****p* value <0.0005).

**Table S4 Effect of sub-MICs of different antimicrobial agents on the viable cell count of S. aureus biofilms according to the SPM.**

| Isolate ID | azithromycin | ciprofloxacin | doxycycline | Gentamicin | imipenem |
| --- | --- | --- | --- | --- | --- |
| *staph1* | 12.5% N.S.  25% N.S.  50% sub-MIC;  **** | N.S. | N.S. | N.S. | N.S. |
| *staph2* | 12.5% sub-MIC;  25% sub-MIC;  ****  50% sub-MIC;  ***** | 12.5% N.S.  25% sub-MIC;  ****  50% N.S. | N.S. | N.S. | 12.5% sub-MIC;  ****  25% sub-MIC;  ***  50% N.S. |
| *staph3* | N.S. | N.S. | N.S. | 12.5% sub-MIC;  ***  25% N.S.  50% N.S. | N.S. |
| *staph4* | N.S. | N.S. | 12.5% N.S.  25% N.S.  50% sub-MIC;  *** | N.S. | N.S. |
| *staph5* | N.S. | N.S. | N.S. | 12.5% sub-MIC;  25% sub-MIC;  ***  50% sub-MIC;  **** | 12.5% sub-MIC;  25% N.S.  50% sub-MIC;  *** |

Each experiment was performed in triplicate. Statistical analysis was performed by paired t tests. N.S. = not significant. The number of asterisks indicates the significance level (**p* value < 0.05, ***p* value <0.005, ****p* value <0.0005).


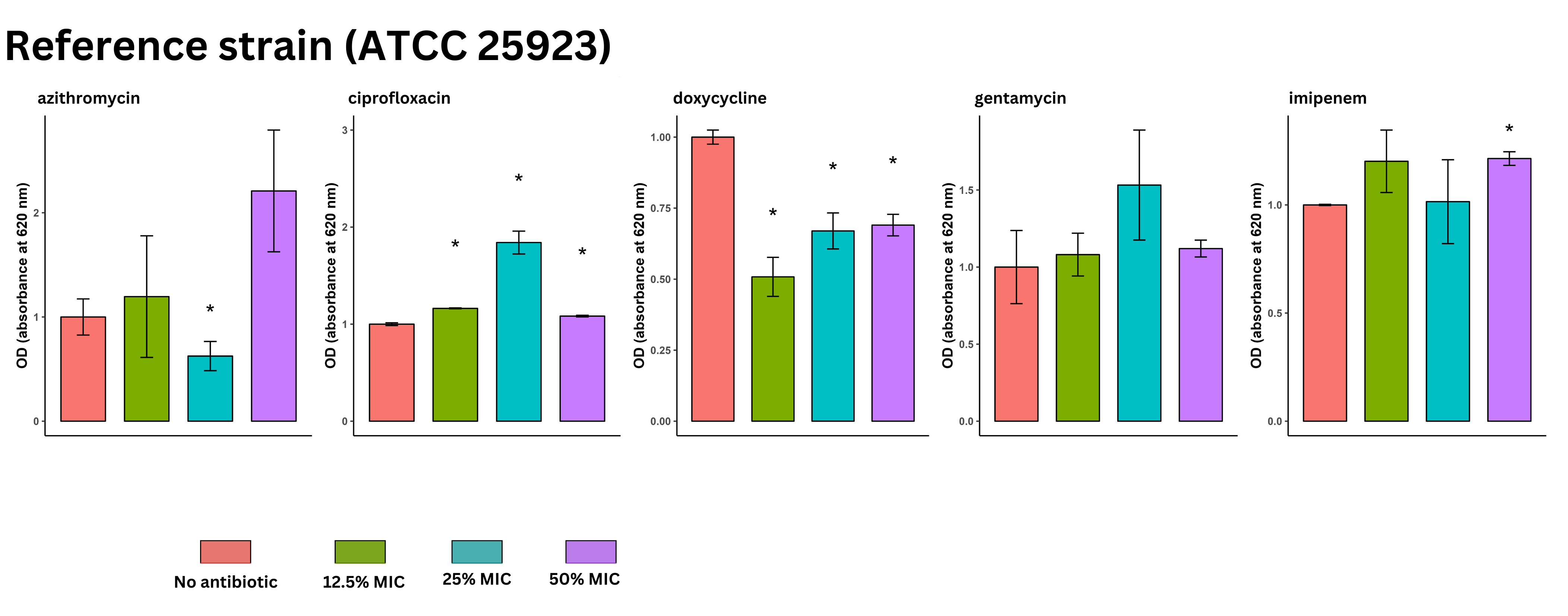


**Figure S 1** Bar plots with error bars (mean ± SD) of CFU count of S. aureus isolates (ATCC 25923) by CV method at baseline (no antimicrobial) and at 3 different sub-MICs (12.5%, 25% and 50%) of azithromycin, ciprofloxacin, doxycycline, gentamicin and imipenem, respectively. All data were normalized to their mean baseline (no-antimicrobial). *Asterisks indicate the statistical significance obtained by paired t-test. The number of asterisks indicates the significance level (*p value < 0.05, **p value <0.005, ***p value <0.0005).


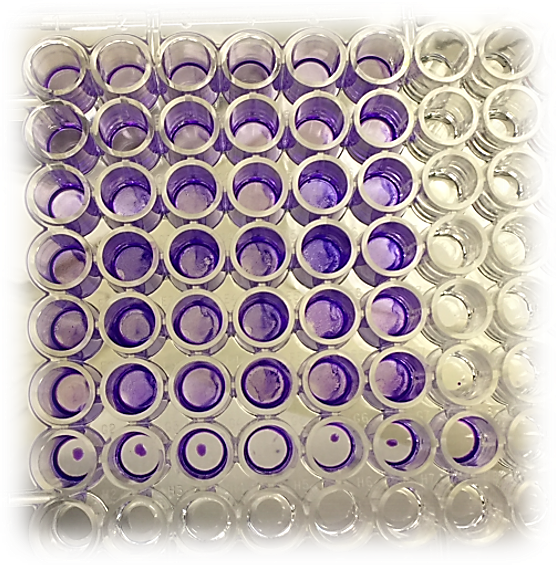


**Figure S 2** A representative figure showing the biofilm formed stained with CV stain


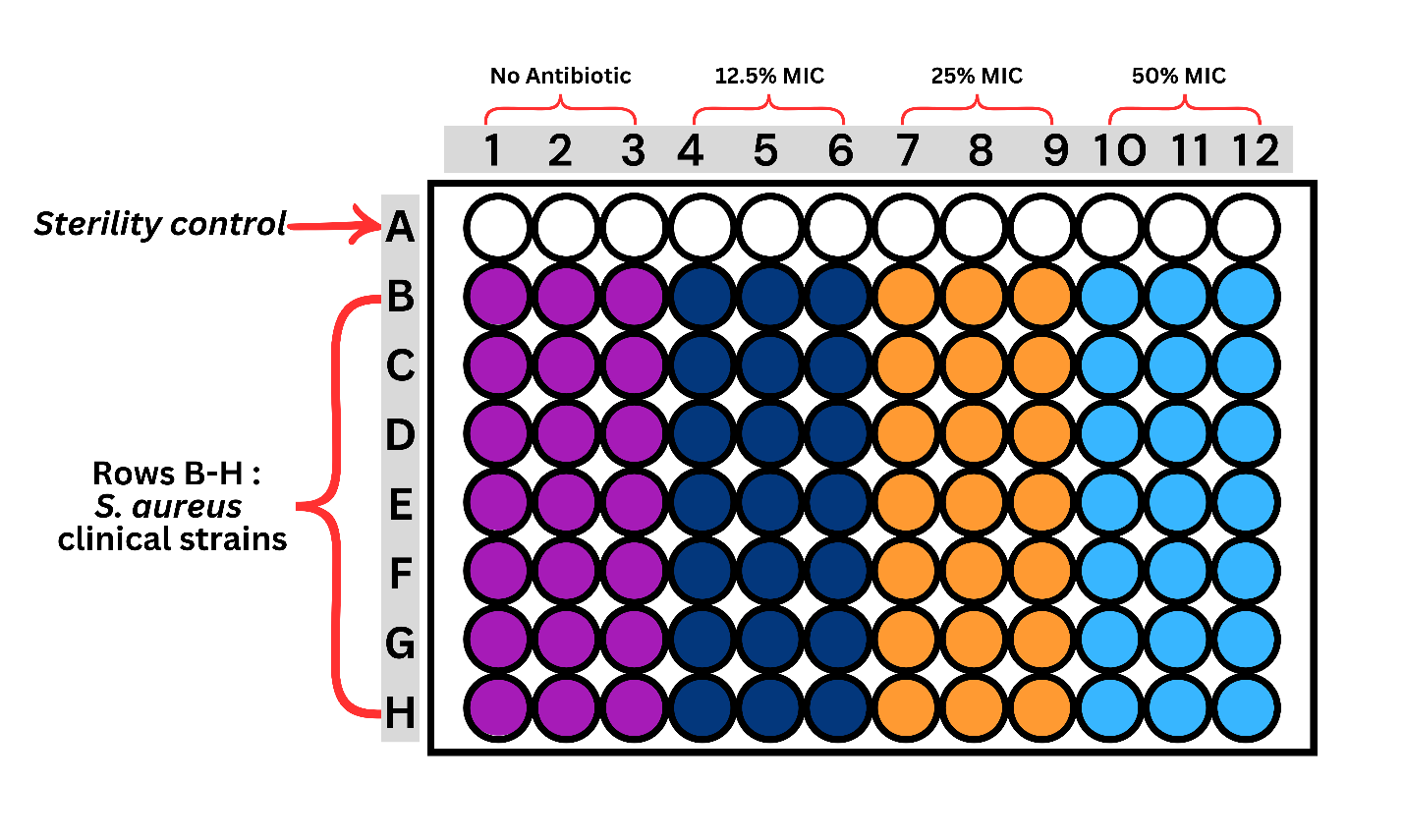


**Figure S 3** Well layout on microplates for Biofilm Formation assay

**Figure S 4** Standard curve created by qPCR using a serial 10fold dilutions of 0.5 McFarland and 16s rRNA primers pair showing the threshold cycle (CT) on Y-axis against the S. aureus bacterial count in CFU on the X-axis.


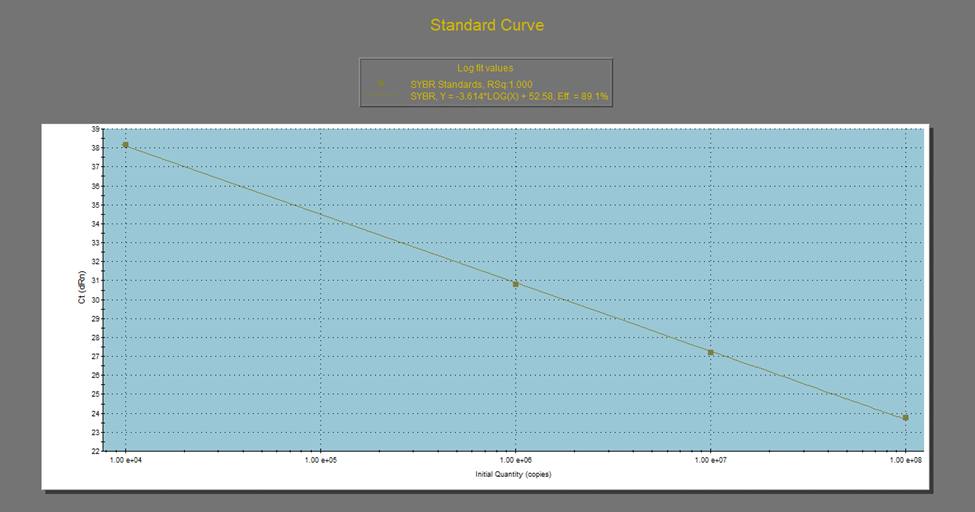


**Figure S 5** The melting curve for some of the results to obtain CT values to be compared with the standard curve to determine the relative amount of DNA.


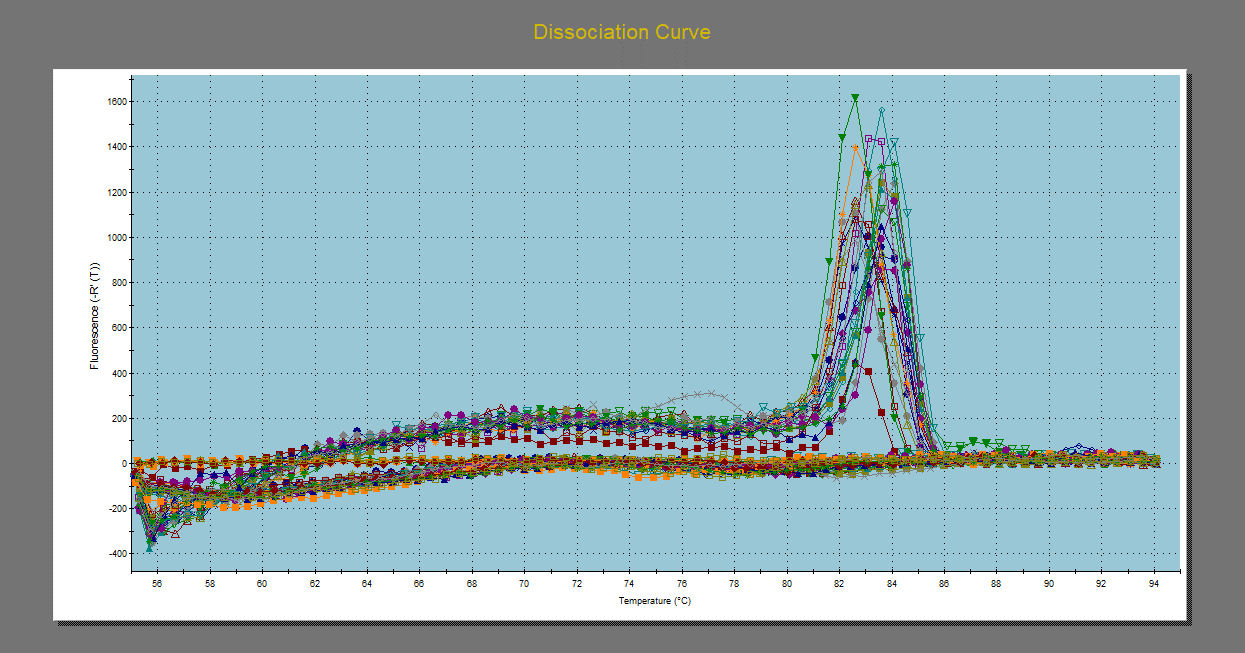

Supplement: Supplementary file 1 — Supplementary Material 1 [file 12879_2024_9790_MOESM1_ESM.docx]
